# Transperineal versus transrectal prostate biopsy in the diagnosis of prostate cancer: a systematic review and meta-analysis

**DOI:** 10.1186/s12957-019-1573-0

**Published:** 2019-02-13

**Authors:** Jianjian Xiang, Huaqing Yan, Jiangfeng Li, Xiao Wang, Hong Chen, Xiangyi Zheng

**Affiliations:** 1 Department of Ultrasound, The First Affiliated Hospital, College of Medicine, Zhejiang University, Hangzhou, China; 2 Department of Urology, The First Affiliated Hospital, College of Medicine, Zhejiang University, Hangzhou, China

**Keywords:** Transperineal, Transrectal, Prostate biopsy, Diagnosis accuracy, Complication

## Abstract

**Background:**

Because conventional prostate biopsy has some limitations, optimal variations of prostate biopsy strategies have emerged to improve the diagnosis rate of prostate cancer. We conducted the systematic review to compare the diagnosis rate and complications of transperineal versus transrectal prostate biopsy.

**Main body of the abstract:**

We searched for online publications published through June 27, 2018, in PubMed, Scopus, Web of Science, and Chinese National Knowledge Infrastructure databases. The relative risk and 95% confidence interval were utilized to appraise the diagnosis and complication rate. The condensed relative risk of 11 included studies indicated that transperineal prostate biopsy has the same diagnosis accuracy of transrectal prostate biopsy; however, a significantly lower risk of fever and rectal bleeding was reported for transperineal prostate biopsy. No clue of publication bias could be identified.

**Short conclusion:**

To conclude, this review indicated that transperineal and transrectal prostate biopsy have the same diagnosis accuracy, but the transperineal approach has a lower risk of fever and rectal bleeding. More studies are warranted to confirm these findings and discover a more effective diagnosis method for prostate cancer.

**Electronic supplementary material:**

The online version of this article (10.1186/s12957-019-1573-0) contains supplementary material, which is available to authorized users.

## Background

Prostate cancer (PCa) is the most common cancer in men and has the second highest mortality in the USA [1]. In 2018, approximately 164,690 PCa cases were identified, accounting for almost one in five new cancer diagnoses [1]. Although PCa is common worldwide, the detection method and diagnostic technology has remained controversial. Generally, the following two significant problems about PCa diagnosis must be settled urgently: (a) prostate-specific antigen (PSA) has been widely adopted for screening PCa; however, the conventional threshold for biopsy (4.0 ng/ml) has been associated with a positive predictive value of approximately 20–30% [2, 3]. Thus, a great number of patients underwent an unnecessary prostate biopsy. Are there better biomarkers to help physicians make biopsy decisions? (b) In 1989, Hodge et al. first reported the systematic sextant prostate biopsy to detect PCa by transrectal ultrasonography (TRUS) guidance [4]. Since then, TR systematic prostate biopsy has been the most valuable technology for diagnosing PCa [5]. Conventional prostate biopsy does have some limitations including severe complications and high rate of false negatives. Therefore, prostate biopsy strategies including guidance technology, biopsy approaches, and number of cores have emerged to improve the diagnosis rate of PCa [6–11]. An urgent need to identify the most effective and safe way to diagnose PCa still remains.

There are the two principle approaches for the diagnosis of PCa: the transperineal (TP) biopsy and the transrectal (TR) biopsy. The systematic TR prostate biopsy, which is the gold standard for the detection of PCa, has been conducted for decades worldwide. This method, however, reportedly underestimates PCa incidence with a false negative rate up to 49% [12]. Additionally, TR prostate biopsy has been reported to cause severe complications such as rectal bleeding, fever, sepsis, hematuria, and acute urinary retention [13–15]. Due to the high false negative and complication rates of the systematic TR prostate biopsy, the TP approach was introduced to improve the detection rate and safety of prostate biopsy. Though a number of studies were carried out to compare the detection rate and complications of the TP and TR prostate biopsy approaches, the results were controversial regarding the detection rate of the two approaches [16–19]. This controversy was mainly on account of the shortage of sample size and insufficient study design. For instance, the study by Tewes et al. reported cancer detection rates of 39% for TR and 75% for TP [10]; however, the study included only 154 patients and the retrospective study design led to a relatively low comparability of the two cohorts. Therefore, the conclusion of the study was not convincing. Without the limitations of observational studies, randomized controlled trials (RCTs) represent the gold standard methodology for clinical studies but the results of several RCTs were also inconsistent [20–23].

Meta-analysis could merge the evidence provided by observational studies and RCTs. To this end, we could not only attain the most extensive study population but also minimize the impact of methodological heterogeneity of each study and eliminate low-quality studies [24]. A meta-analysis that offers a higher level of evidence is needed to draw a reliable conclusion about the two biopsy approaches. Previous meta-analyses simply merged observational studies and RCTs together, which brought methodological heterogeneity to the analyses because the study designs and quality assessment methods differed [8]. Aiming to achieve a more precise and convincing conclusion about the detection rates and complications of TP and TR approaches, we separately synthesized observation studies and RCTs after a strict study quality assessment. Additionally, we systematically reviewed all eligible studies to compare the complications of the two biopsy methods.

## Materials and methods

### Literature search

Our review was conducted on the basis of the PRISMA guidelines [25]. We searched for literatures in PubMed, Scopus, Web of Science, and Chinese National Knowledge Infrastructure (CNKI) databases to cover publications through June 27, 2018 [24, 26]. We utilized a robust and comprehensive retrieval strategy including phrases of two approaches (perineal or transperineal) and (rectal or transrectal). Then, we assessed the obtained papers by looking through their headings and abstracts. Every single potentially relevant study that matched our inclusion requirements was included. The reference documents of the included articles were also completely reviewed to detect any other related study. The language was restricted to English and Chinese. The literature retrieving was performed by two authors solely, and disagreement was settled by consensus.

### Inclusion criteria

For the studies contained in our review, all of the subsequent undermentioned criteria should be met: (1) they were designed to be an RCT study, cohort study, or case-control study. (2) The subjects of the studies comprised patients who underwent prostate biopsy. (3) The intervention method included the transperineal approach and the transrectal approach. (4) Apart from the biopsy approach, the number of cores and the guidance method remained the same. (5) The final outcome of the cases included a diagnosis of PCa or complications of the two approaches. (6) The studies provided odds ratios (ORs) or relative risks (RRs) with their 95% confidence intervals (CIs), or adequate evidence to estimate them [26].

### Data extraction

The data from the included studies was separately condensed by two authors utilizing a predetermined statistics table and any disagreement was settled by discussion. The crucial aspects were assembled from the included studies: the first author’s last name and country, the publication year, the age of the patients, study design, study population, the number of patients in the two groups, the PSA level and prostate volume of the patients, biopsy methods, and the covariates in the analyses. For respective study, we alternatively extracted the RR or OR which were adjusted for the largest number of confounders [26]. If no RR or OR could be extracted for the whole study, we extracted the original data and calculated the raw RR or OR to estimate the diagnosis accuracy of the two approaches.

### Quality assessment

Two authors separately conducted the quality assessment of studies based on the Newcastle–Ottawa scale (NOS) applied for observational studies with Cochrane Tool Review Manager 5.3 for RCTs. Observational studies with a < 7 NOS score were defined as low quality and excluded. For RCTs, only one RCT by Udeh et al. was excluded because one in four patients was lost follow-up, representing a high attrition bias [27]. Disagreements between the authors were settled by consensus. If no agreement was achieved, another third expert was invited to resolve the problem.

### Statistical methods

As all included studies were cohort studies and RCTs, RR was used to estimate the diagnosis accuracy of TP and TR approaches. If the paper did not contain an adjusted RR and its 95% CI, the initial data was extracted to estimate the raw RR and its 95% CI. We synthesized the RRs and their matching 95% CIs by a random effect model because this model considers the variation both inside the study and between the study [28]. The heterogeneity between the studies was determined by the *Q* test and *I*^2^, as a quantification of heterogeneity, simultaneously calculated to precisely demonstrate the scale of heterogeneity. If significant heterogeneity was detected (*I*^2^ > 50%), a systematic review would be conducted instead of a meta-analysis. As the number of patients with complications was zero in some studies, the complications after prostate biopsy were systematically reviewed rather than calculating the overall RR. Publication bias was checked utilizing Begg’s test and Egger’s test, and *P* < 0.05 was defined to indicate a significant publication bias for the meta-analysis [29, 30]. The stability of the attained results was checked by sensitivity analyses. We deleted a lone study each time to reveal the impact of a particular study to the merged RR.

## Results

### Literature search

**Fig. 1:**
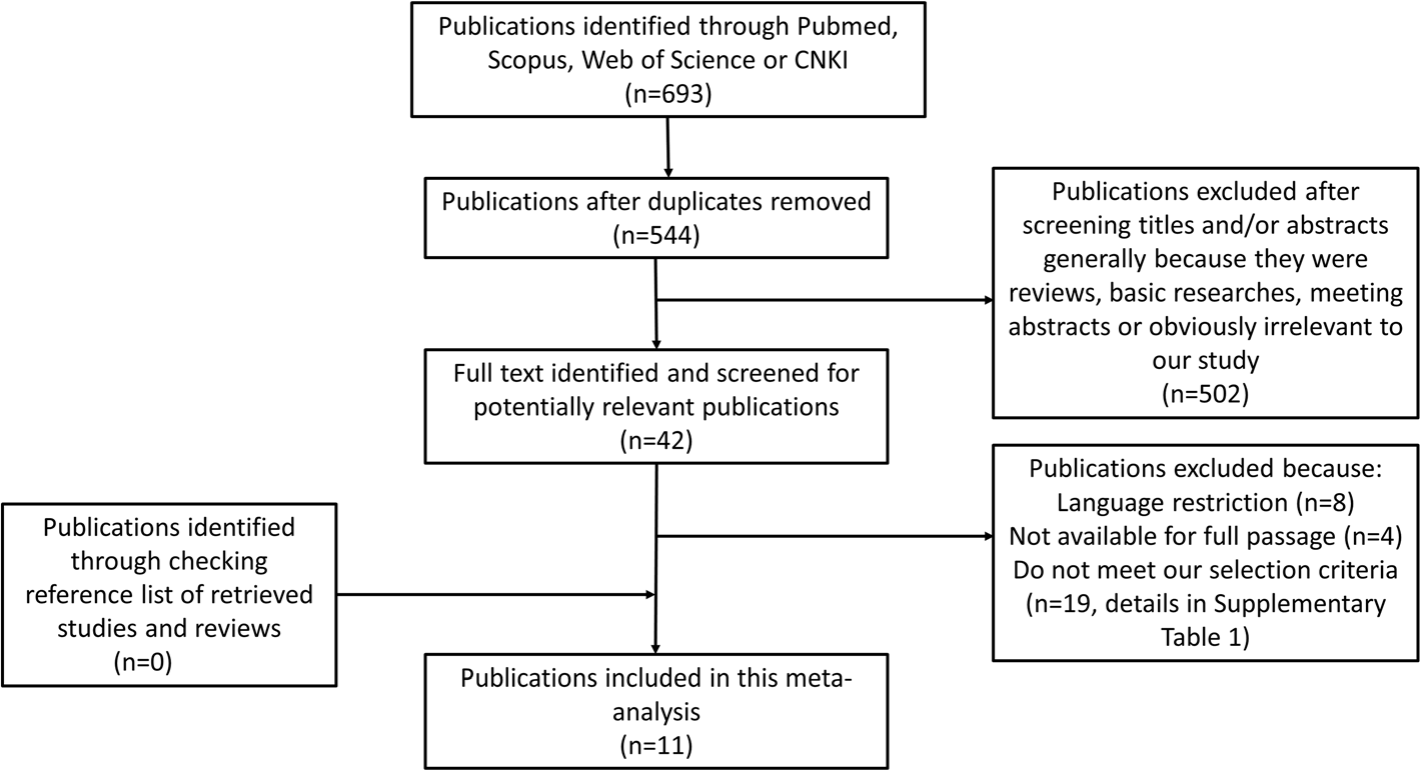
Flowchart of study assessment and selection

Figure 1 details our retrieval and selection and collection process. Briefly, 544 publications were identified after duplicates were removed. Of these, a large portion of the publications was excluded after scanning the headings and abstracts because they were reviews, fundamental research, meeting abstracts, or extraneous to our study. Next, we identified and carefully reviewed 42 potentially relevant publications of which 31 studies were excluded for language restriction, not available for full passage or not meeting our selection criteria (details in Additional file 1: Table S1). No publications were obtained by trailing through the references of the included articles. Hence, a total of seven cohort studies and four RCTs meeting the inclusion criteria were included in this meta-analysis [13, 16–23, 31, 32].

### Study characteristics

We displayed the components of the seven observational studies in Table 1 and four RCTs in Table 2. The study population in the 11 studies was from Italy, China, and Japan. All the included studies were reported between 2002 and 2017. The sample volume fluctuated from 107 [16] to 402 [32]. The total population included in this meta-analysis reached 2569 with 1644 for the TP approach and 1634 for the TR approach (study by Emiliozzi et al., Pepe et al., and Watanabe et al. were performed with a self-control method). More than two potential confounding factors were adjusted in all observational studies.

**Table 1:** Study characters of RCTs comparing TP and TR prostate biopsy

Study	Age	Study population	Patients		PSA level		Prostate volume		Biopsy methods
			TP group	TR group	TP group	TR group	TP group	TR group	
Hara et al., 2008, Japan [21]	71	Patients with a PSA level of 4.0 to 20.0 ng/mL from 2003.5 to 2005.10	126	120	8.34	8.48	33.2	36	Systematic 12-core biopsy
Takenaka et al., 2008, Japan [22]	71 in TP group, 72 in TR group	Consecutive patients with an elevated PSA level (> 4 ng/mL)	100	100	17.1	19.6	34.5	37.2	Systematic 12-core biopsy
Cerruto et al., 2014, Italy [23]	66.5 in TP group, 67.3 in TR group	Consecutive patients with a PSA > 4 ng/mL	54	54	15.95	12.36	56.29	61.49	Systematic 14-core initial prostatic biopsy
Guo et al., 2015, China [20]	67	Patients between 2012.6 and 2014.8 with a PSA > 4.0 ng/ml	173	166	8.81	10.48	47.2	45.9	Systematic 12-core biopsy

*Abbreviations*: *TP* transperineal, *TR* transrectal, *PSA* prostate-specific antigen

**Table 2:** Study characters of observational studies comparing TP and TR prostate biopsy

Study	Age	Study design	Study population	Patients		PSA level		Prostate volume		Biopsy methods	Covariates	NOS score
				TR group	TP group	TR group	TP group	TR group	TP group			
Emiliozzi et al., 2002, Italy [16]	68	Cohort study	Patients with a PSA > 4 ng/ml between 2000.4 and 2001.5	107		8.2		NA		Six transperineal cores plus six transrectal cores	Self-control	9
Watanabe et al., 2005, Japan [32]	72.5	Cohort study	Patients with clinically suspicious prostatic irregularities between 1995.1 and 2001.12	402		10.3		NA		Combined 6-core transperineal and 6-core transrectal biopsies	Self-control	9
Abdollah et al., 2010, Italy [19]	66.3	Cohort study	Patients who underwent a rebiopsy between 2005.9 and 2008.6	140	140	9.7	10	65.4	62.3	Ultrasound-guided saturate prostate rebiopsy	Age, PSA, PV, DRE, histologic findings on previous biopsy, the number of previous negative biopsy sets	8
Tian et al., 2014, China [31]	63 in TP group, 64 in TR group	Cohort study	Patients who underwent a biopsy between 2007.8 and 2012.7	175	137	1.91–112.52	1.45–108.27	59.5	62.4	Ultrasound-guided systematic prostate biopsy	Age, PSA, DRE findings, PV	7
Yuan et al., 2014, China [13]	66	Cohort study	Patients who underwent a biopsy between 2009.1 and 2014.1	59	97	21.2	19.7	33.7	35.8	Ultrasound-guided systematic prostate biopsy	Age, PSA, PV	7
Pepe et al., 2016, Italy [18]	61	Cohort study	Patients persistently suspicious of PCa between 2015.1 and 2016.1	200		8.6		NA		mpMRI/TRUS fusion-targeted biopsy	Self-control	9
Franco et al., 2017, Italy [17]	68 in TP group, 66 in TR group	Cohort study	Random patients that received a prostate biopsy between 2004 and 2014	108	111	7.8	6.9	NA		Ultrasound-guided systematic sextant prostate biopsy	Age, PSA, PSA ratio (F/T), DRE/TRUS findings, LUTS, BPH, biopsy cores, complications	7

*Abbreviations*: *TP* transperineal, *TR* transrectal, *PSA* prostate-specific antigen, *PV* prostate volume, *DRE* digital rectal examination, *PSA ratio F/T* free PSA/total PCA, *TRUS* transrectal ultrasonography, *LUTS* lower urinary tract syndrome, *BPH* benign prostate hyperplasia, *NA* not available

### Data obtained from RCTs

**Fig. 2:**
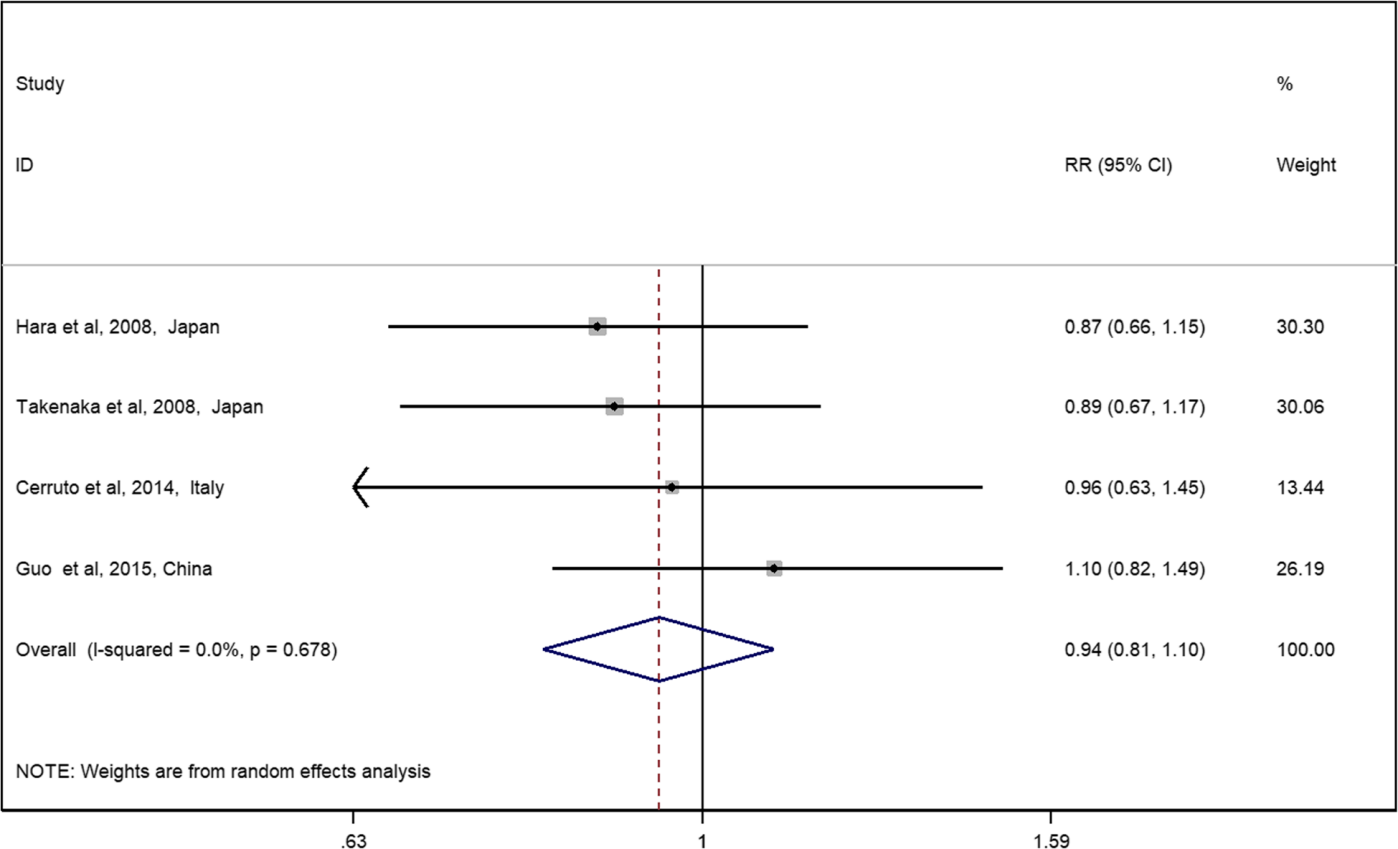
Relative risks for RCTs assessing the diagnosis rate of the TP approach vs the TR approach. Notes: diamonds represent study-specific relative risks (RRs) or summary relative risks with 95% confidence intervals (CIs). Horizontal lines represent 95% CIs. Test for heterogeneity among studies: *P* = 0.678, *I*^2^ = 0.0%

The general RR and its 95% CI showed no significant difference between the TP and TR approaches on diagnosis accuracy (Fig. 2, RR 0.94, 95% CI 0.81–1.10). No significant heterogeneity was detected among these studies with *Q* = 1.52, *I*^2^ = 0%, and *P* = 0.678. Generally, all RCTs were assessed to have a low risk of bias (Additional file 1: Figure S1). The performance bias was high in all studies because blinding patients with biopsy approach is not possible; however, in this study, performance bias may not affect the accuracy of the results.

### Data obtained from observational studies

**Fig. 3:**
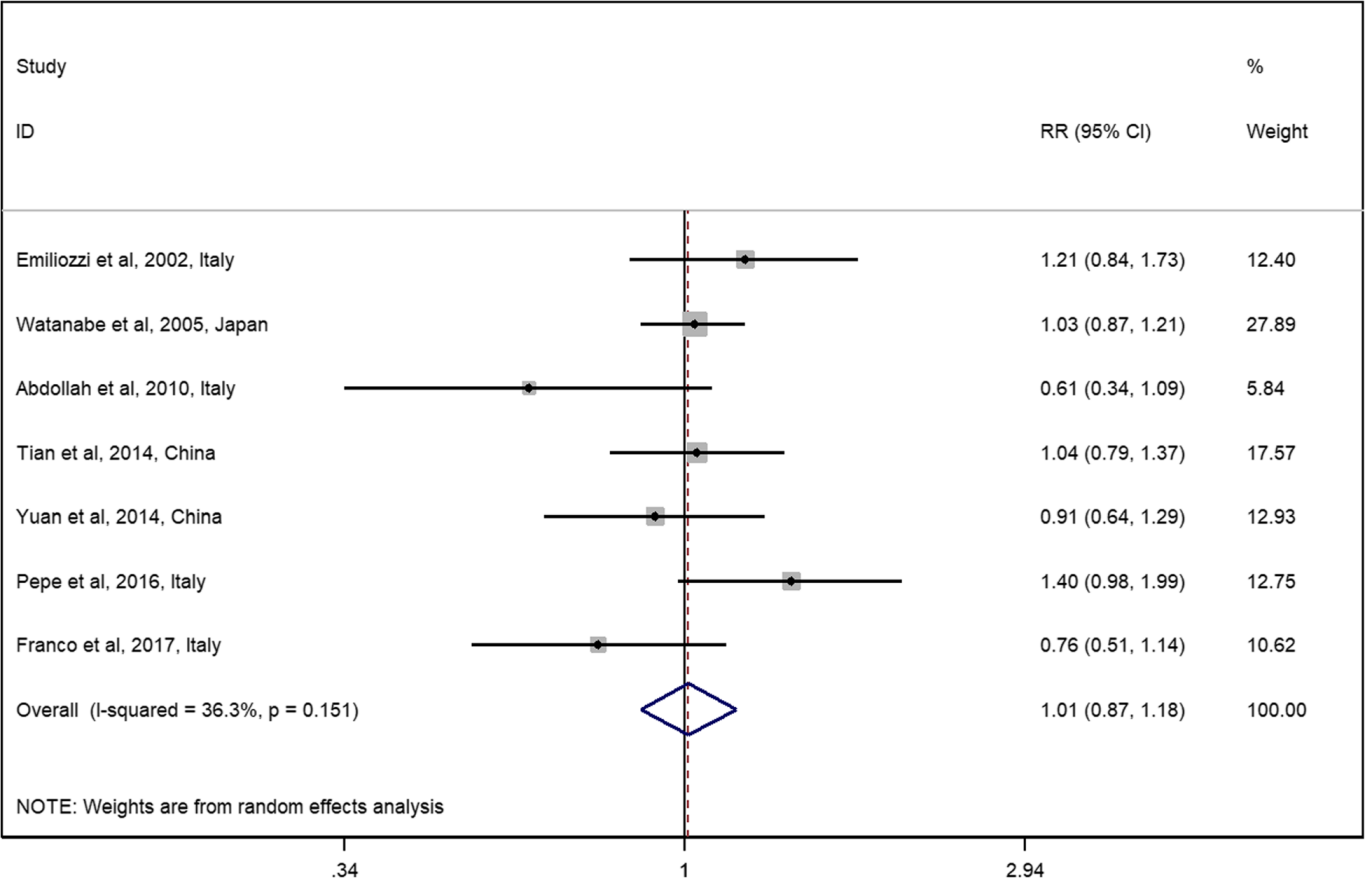
Relative risks for observational studies assessing the diagnosis rate of the TP approach vs the TR approach. Notes: diamonds represent study-specific relative risks (RRs) or summary relative risks with 95% confidence intervals (CIs). Horizontal lines represent 95% CIs. Test for heterogeneity among studies: *P* = 0.151, *I*^2^ = 36.3%

The general RR and its 95% CI showed no significant difference between the TP and TR approaches on diagnosis accuracy (Fig. 3, RR 1.01, 95% CI 0.87–1.18), which is consistent with the results of the RCTs. No significant heterogeneity was detected among the observational studies (*Q* = 9.42, *I*^2^ = 36.3%, and *P* = 0.151). All included observational studies were assessed to be of high quality (NOS score > 6).

### Comparison of complications of the two approaches

As every RR for each complication was not available, we systematically reviewed all studies comparing the complications of the two approaches. The detailed number of patients with complications is shown in Table 3. In addition, we calculated the RR of each complication using the synthesized data. The TP approach significantly protected the patients from rectal bleeding (RR = 0.02, 95% CI 0.01–0.06) and fever (RR = 0.26, 95% CI 0.14–0.28); however, the TP approach significantly increased patient pain (RR = 1.83, 95% CI 1.27–2.65). No significant difference was found in the acute retention of urine and hematuria between the two approaches.

**Table 3:** Comparison of complications of TP and TR prostate biopsy

Study	Total population		Rectal bleeding		Acute retention of urine		Hematuria		Fever		Pain	
	TP	TR	TP	TR	TP	TR	TP	TR	TP	TR	TP	TR
Hara et al., 2007, Japan [21]	126	120	0	0	2	3	13	11	0	2	NA	
Takenaka et al., 2008, Japan [22]	100	100	0	1	2	3	11	12	1	2	NA	
Tian et al., 2014, China [31]	175	137	0	7	10	8	12	11	6	13	16	11
Yuan et al., 2014, China [13]	59	97	2	49	4	7	25	53	2	15	NA	
Cerruto et al., 2014, Italy [23]	54	54	0	4	0	1	5	0	0	1	NA	
Guo et al., 2015, China [20]	173	166	0	16	NA		33	37	2	9	58	26
Franco et al., 2017, Italy [17]	125	132	0	4	2	3	3	3	NA		0	3
Total Number	812	806	2	81	20	25	102	127	11	42	74	40
RR (95% CI), TR as the control group	/		0.02 (0.01–0.06)		0.89 (0.50–1.59)		0.79 (0.63–1.01)		0.26 (0.14–0.48)		1.83 (1.27–2.65)	

*Abbreviations*: *TP* transperineal, *TR* transrectal, *RR* relative risk, *NA* not available

### Sensitivity analysis

To confirm the stability of the merged results, a sensitivity analysis of the integrated RRs was conducted. Based on the random effects model, the general RRs were once again calculated through discarding every single study in the meta-analysis. As a result, the RRs (Additional file 1: Tables S2-S3) persist constantly.

### Publication bias

**Fig. 4:**
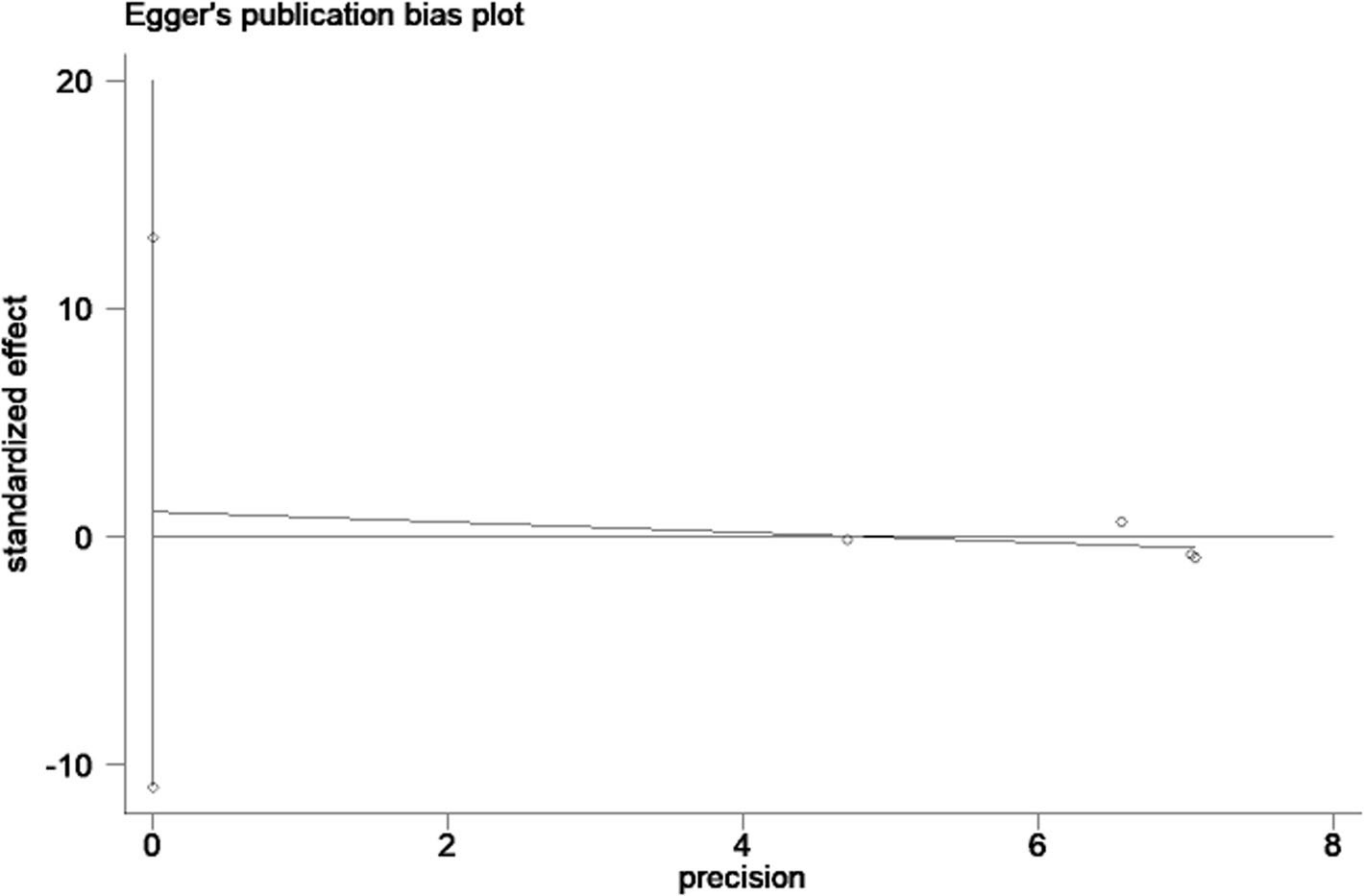
Egger’s publication bias plot for RCTs. Notes: Egger’s regression asymmetry test (*P* = 0.74). Standardized effect was defined as the odds ratio divided by its standard error. Precision was defined as the inverse of the standard error

**Fig. 5:**
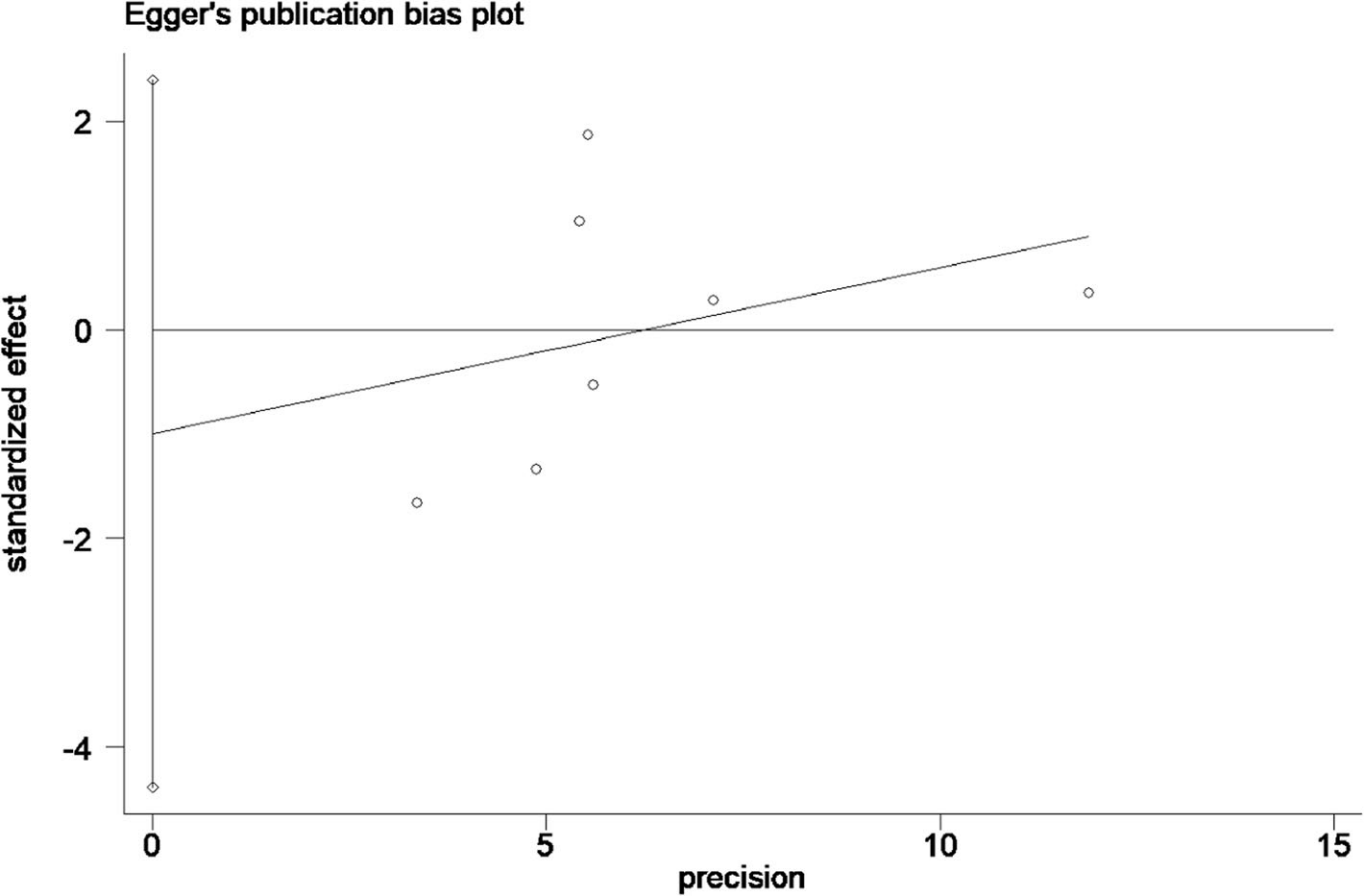
Egger’s publication bias plot for observational studies. Notes: Egger’s regression asymmetry test (*P* = 0.49). Standardized effect was defined as the odds ratio divided by its standard error. Precision was defined as the inverse of the standard error

For the RCTs, neither Begg’s test (*P* = 0.31) nor Egger’s test (Fig. 4, *P* = 0.74) demonstrated a significant publication bias. Similarly, for the observational studies, publication bias was not significant upon Begg’s test (*P* = 0.37) or Egger’s test (Fig. 5, *P* = 0.49).

### Comparison of MRI/US fusion-guided biopsy with systematic transrectal biopsy

Emerging evidence has shown that multiparametric magnetic resonance imaging (mpMRI) as an innovative guidance approach for prostate biopsy increases the detection rate of prostate cancer. Hence, we also reviewed RCT studies comparing MRI/US fusion-guided biopsy and traditional systematic transrectal biopsy. This review was not included in our meta-analysis as our aim was to assess the diagnosis accuracy of transperineal and transrectal biopsy.

Observational studies have limitations in population selection, comparability, and recall bias; however, RCT studies as the gold standard in clinical trial design could significantly avoid known disadvantages. Here, we identified two RCT studies comparing MRI/US fusion-guided transperineal biopsy with systematic transrectal biopsy. Both the studies were assessed to have a low risk of bias. In the study by Baco et al. [33], a total of 175 biopsy-naive patients with suspicion for PCa were randomized into two groups: the MRI group (*n* = 86) and the control group (*n* = 89). In the MRI group, the patients underwent an MRI/US fusion-guided 2-core biopsy followed by a traditional 12-core transrectal biopsy. In the cases with negative MRI findings, only a 12-core RB was performed. For the patients in the control group, a 2-core targeted biopsy for abnormal DRE/TRUS and 12-core traditional transrectal biopsy were conducted. The authors revealed a comparable detection rate between the 2-core MRI/US fusion biopsy and traditional 12-core systematic transrectal biopsy, suggesting that the traditional systematic transrectal biopsy could be replaced by the transrectal 2-core MRI/US fusion biopsy.

In the other RCT study by Kasivisvanathan et al. [34], the authors randomized 252 patients in an MRI-targeted group and 248 patients in a standard biopsy group. In the MRI-targeted group, 71 patients did not undergo prostate biopsy because of negative MRI results. The patients in the MRI-targeted group received a 4-core MRI/US fusion biopsy and the patients in the standard biopsy group received a systematic transrectal biopsy. Clinically significant prostate cancer was diagnosed in 38% patients in the MRI-targeted group and 26% patients in the standard biopsy group. The detection rate of the MRI-targeted biopsy is significantly higher than the traditional biopsy.

## Discussion

This meta-analysis of seven observational studies and four RCTs indicated that the transperineal prostate biopsy and the transrectal prostate biopsy were similar in diagnosis efficiency. A quantified *Q* test and *I*^2^ test were performed to appraise the intensity of heterogeneity between the studies and showed no significant heterogeneity. We calculated the synthesized RR again using the fixed effect model and the results remained the same (RR = 1.02, 95% CI 0.92–1.14 for observational studies and RR = 0.95, 95% CI 0.81–1.10 for RCTs). The heterogeneity of our study was not significant. Our results remained consistent upon sensitivity analysis, indicating that our results are stable and reliable. Additionally, no evidence of significant publication bias was detected with either Begg’s test or Egger’s test. These results vastly improved the reliability and certainty of our work. Our results were consistent with previous studies [13, 16–23, 31, 32].

Apart from the detection efficiency of prostate biopsy, complications also play an important role in evaluating the safety and value of the biopsy method. Our study revealed that the TP approach significantly decreased the risk of complications including rectal bleeding and fever, while the TR approach significantly protected patients from pain. The two approaches had no significant difference in acute retention of urine and hematuria. Generally, rectal bleeding and hematuria are self-limited complications and patients would obtain relief within several days; however, bleeding can be severe, especially in patients taking anticoagulation drugs such as aspirin. For these patients, anticoagulation drugs should be withdrawn for at least 1 week prior to undergoing prostate biopsy to avoid severe bleeding events. Infections or fever are also common after prostate biopsy. Though enemas are conducted before the transrectal prostate biopsy, the TR approach still had a significantly higher risk of infection than the TP approach. For patients who are prone to infection including those with diabetes, prostatitis, and urinary catheterization, the transperineal prostate biopsy was recommended to avoid sepsis and severe fever after the procedure. Additionally, transperineal prostate biopsy was more comfortable prior to the biopsy because the enema was unnecessary. Most patients would undertake pain after prostate biopsy. Though our study showed that patients that underwent transperineal prostate biopsy were more likely have pain, it is often diminished within several days [31]. Analgesia drugs could be used in moderation for relieving patients’ pain. On the other hand, the TP approach was confirmed to be superior in detecting tumors in the transitional zone and apex of the prostate [16, 22, 23].

Our study evaluating the diagnosis accuracy of the two approaches was more credible because (a) a clear and powerful approach was taken to search the online database to obtain all potentially relevant publications and obedience to PRISMA guidelines and (b) the most comprehensive studies up to date were included in this study. We utilized a strict inclusion criteria constraint in which only RCTs with a low risk of bias and high-quality cohort studies (defined as NOS score > 6) were included. The RCT by Udeh et al. was excluded because 25% of patients were lost follow-up, indicating a high risk of bias [27]. (c) Unlike previous meta-analyses, we separately synthesized observational studies and RCTs because they have different quality assessment methods and simply pooling these results may reduce the reliability of the meta-analysis [8].

At the same time, some limitations should be mentioned. First, only four RCTs, which represented the gold standard methodology of clinical trials, were included in our study. For cohort studies, the selection and comparability problems could not be avoided. We could not solve the potential confounding factors such as free PSA, benign prostate hyperplasia, or other unreported factors in the included cohort studies. Second, though no significant publication bias could be detected, we could not rule out the possibility that our conclusions may be affected by potential publication bias mainly because of the language limitation and the screening approach that only published studies could be included in our study.

MRI/US fusion biopsy as a novelty for prostate biopsy could significantly reduce the biopsy cores. In light of the RCT by Baco et al., the detection efficiency of 2-core MRI/US fusion biopsy was similar with systematic transrectal biopsy [33]; however, the MRI group in Baco’s trial included patients with negative MRI results that underwent only systematic transrectal biopsy. These patients could reduce the detection rate of MRI/US fusion biopsy. In the other RCT by Kasivisvanathan et al. [34], the authors excluded patients with negative MRI results and detected a significantly higher detection rate upon MRI/US fusion biopsy compared to traditional transrectal biopsy. With these findings, we may conclude that along with improving the biopsy accuracy, MRI might also free patients from an unnecessary prostate biopsy [33–36]. This result was in accordance with our previous review for observational studies in this field [37].

## Conclusion

In conclusion, our study indicated that transperineal prostate biopsy has the same diagnosis accuracy of transrectal prostate biopsy; however, transperineal prostate biopsy is safer and more valuable because it poses a significantly lower risk of infection and rectal bleeding. Despite the increased risk of pain after TP biopsy, we recommend that doctors should perform transperineal prostate biopsy if possible. An MRI should be conducted before a biopsy to avoid an unnecessary prostate biopsy. To the best of our knowledge, a 2–4-core MRI/US fusion-targeted transperineal biopsy may be the best method for prostate biopsy. More studies should be conducted to confirm findings and discover a more effective diagnosis method for prostate cancer.

## Additional file

Additional file 1: **Table S1.** Details of excluded studies. **Table S2.** Sensitivity analysis of RCTs. **Table S3.** Sensitivity analysis of observational studies. **Figure S1.** Risk of bias assessment of RCTs. (ZIP 605 kb)

## Abbreviations

BPH: Benign prostate hyperplasia
CI: Confidence interval
DRE: Digital rectal examination
LUTS: Lower urinary tract syndrome
mpMRI: Multiparametric magnetic resonance imaging
NA: Not available
NOS: Newcastle–Ottawa scale
OR: Odds ratio
PCa: Prostate cancer
PRISMA: Preferred Reporting Items for Systematic Reviews and Meta-analyses
PSA ratio F/T: Free PSA/total PSA
PSA: Prostate-specific antigen
PV: Prostate volume
RCT: Randomized controlled trial
RR: Relative risk
TP: Transperineal
TR: Transrectal
TRUS: Transrectal ultrasonography
US: Ultrasonography
