# Supplementary material for: Transperineal versus transrectal prostate biopsy in the diagnosis of prostate cancer: a systematic review and meta-analysis
Source: World J Surg Oncol. 2019 Feb 13;17:31. doi: 10.1186/s12957-019-1573-0 (PMC6375152; doi:10.1186/s12957-019-1573-0)
Supplement: Supplementary file 1 — Table S1. Details of excluded studies. Table S2. Sensitivity analysis of RCTs. Table S3. Sensitivity analysis of observational studies. Figure S1. Risk of bias assessment of RCTs. (ZIP 605 kb) [file 12957_2019_1573_MOESM1_ESM.zip › Supplementary data.docx]

**Supplementary Table 1.** Details of excluded studies.

| Study | Reason |
| --- | --- |
| Hossack et al, 2012, Australia[^1^](#_ENREF_1) | The study was conducted in patients who underwent radical prostatectomy |
| Vis et al, 2000, Netherland[^2^](#_ENREF_2) | The study was conducted in patients who underwent radical prostatectomy |
| Nafie et al, 2017, UK[^3^](#_ENREF_3) | The study compared 36-core transperineal biopsy with 12-core transrectal biopsy |
| Dimmen et al, 2012, Norway[^4^](#_ENREF_4) | The study was conducted in patients with negative transrectal biopsy |
| Huang et al, 2016, China[^5^](#_ENREF_5) | The study compared traditional 12-core transrectal biopsy with image fusion guided transperineal biopsy |
| Borkowetz et al, 2015, Germany[^6^](#_ENREF_6) | The study compared systematic transrectal biopsy with MRI/US guided transperineal biopsy |
| Ong et al, 2015, Australia[^7^](#_ENREF_7) | The study had no referred group |
| Scott et al, 2015, Australia[^8^](#_ENREF_8) | The study was conducted in patients who underwent radical prostatectomy |
| Udeh et al, 2015, Nigerian[^9^](#_ENREF_9) | The RCT was assessed to be high risk of bias for high detection bias (outcome assessment was not blinded) and attrition bias (25% patients lost follow-up) |
| Krughoff et al, 2013, US[^10^](#_ENREF_10) | The study compared ultrasound-guided transrectal biopsy with transperineal 3-dimensional mapping biopsy |
| Kawakami et al, 2006, Japan[^11^](#_ENREF_11) | The study compared conventional transrectal sextant biopsy with TRUS-guided systematic three-dimensional 26-core biopsy |
| Shinda et al, 2016, Japan[^12^](#_ENREF_12) | The study compared a combined transrectal and transperineal approach with a transrectal approach for prostate rebiopsy |
| Whelan et al, 1986, Canada[^13^](#_ENREF_13) | Without sufficient data |
| Grummet et al, 2014, Australia[^14^](#_ENREF_14) | The study had no referred group |
| Miller et al, 2005, Australia[^15^](#_ENREF_15) | The study cohorts are of low comparability (the most important factors including age, PSA and abnormal DRE rate were significantly different) |
| Dieffenbacher et al, 2017, Germany[^16^](#_ENREF_16) | The study compared transperineal MRI/TRUS fusion prostate biopsy vs. transrectal prostate biopsy |
| Tewes et al, 2017, Germany[^17^](#_ENREF_17) | The study cohorts are of low comparability (the most important factors including age, PSA, PI-RADS score were significantly different) |
| Namekawa et al, 2015, Japan[^18^](#_ENREF_18) | The study had no referred group |
| Miano et al, 2014, Italy[^19^](#_ENREF_19) | Without sufficient data |

**Supplementary Table 2.** Sensitivity analysis of RCTs.

| **Study omitted** | **RR (95% CI)** | **Heterogeneity test** | | |
| --- | --- | --- | --- | --- |
|  |  | **Q** | **P** | **I^2^(%)** |
| Hara et al, 2008, Japan[^20^](#_ENREF_20) | 0.98 (0.81-1.17) | 1.04 | 0.59 | 0 |
| Takenaka et al, 2008, Japan[^21^](#_ENREF_21) | 0.97 (0.81-1.16) | 1.27 | 0.53 | 0 |
| Cerruto et al, 2014, Italy[^22^](#_ENREF_22) | 0.94 (0.80-1.11) | 1.51 | 0.47 | 0 |
| Guo et al, 2015, China[^23^](#_ENREF_23) | 0.89 (0.75-1.07) | 0.15 | 0.93 | 0 |

**Supplementary Table 3.** Sensitivity analysis of observational studies.

| **Study omitted** | **RR (95% CI)** | **Heterogeneity test** | | |
| --- | --- | --- | --- | --- |
|  |  | **Q** | **P** | **I^2^(%)** |
| Emiliozzi et al, 2002, Italy[^24^](#_ENREF_24) | 0.98 (0.83-1.17) | 8.49 | 0.13 | 41.1 |
| Watanabe et al, 2005, Japan[^25^](#_ENREF_25) | 1.00 (0.81-1.23) | 9.40 | 0.09 | 46.8 |
| Abdollah et al, 2010, Italy[^26^](#_ENREF_26) | 1.04 (0.91-1.19) | 6.29 | 0.28 | 20.5 |
| Tian et al, 2014, China[^27^](#_ENREF_27) | 1.00 (0.83-1.21) | 9.40 | 0.09 | 46.8 |
| Yuan et al, 2014, China[^28^](#_ENREF_28) | 1.03 (0.86-1.22) | 8.95 | 0.11 | 44.1 |
| Pepe et al, 2016, Italy[^29^](#_ENREF_29) | 0.98 (0.85-1.12) | 6.07 | 0.30 | 17.6 |
| Franco et al, 2017, Italy[^30^](#_ENREF_30) | 1.05 (0.90-1.21) | 7.16 | 0.21 | 30.2 |

**Figure legends**

**Figure S1.** Risk of bias assessment of RCTs.

Abbreviation: RCT, randomized controlled trial.

**References**

1. Hossack T, Patel MI, Huo A, et al. Location and pathological characteristics of cancers in radical prostatectomy specimens identified by transperineal biopsy compared to transrectal biopsy. The Journal of urology 2012;188:781-5.

2. Vis AN, Boerma MO, Ciatto S, Hoedemaeker RF, Schroder FH, van der Kwast TH. Detection of prostate cancer: a comparative study of the diagnostic efficacy of sextant transrectal versus sextant transperineal biopsy. Urology 2000;56:617-21.

3. Nafie S, Wanis M, Khan M. The efficacy of transrectal ultrasound guided biopsy versus transperineal template biopsy of the prostate in diagnosing prostate cancer in men with previous negative transrectal ultrasound guided biopsy. Urology Journal 2017;14:3008-12.

4. Dimmen M, Vlatkovic L, Hole KH, Nesland JM, Brennhovd B, Axcrona K. Transperineal prostate biopsy detects significant cancer in patients with elevated prostate-specific antigen (PSA) levels and previous negative transrectal biopsies. BJU international 2012;110:E69-E75.

5. Huang H, Wang W, Lin T, et al. Comparison of the complications of traditional 12 cores transrectal prostate biopsy with image fusion guided transperineal prostate biopsy. BMC urology 2016;16:1-6.

6. Borkowetz A, Platzek I, Toma M, et al. Comparison of systematic transrectal biopsy to transperineal magnetic resonance imaging/ultrasound-fusion biopsy for the diagnosis of prostate cancer. BJU international 2015;116:873-9.

7. Ong WL, Weerakoon M, Huang S, et al. Transperineal biopsy prostate cancer detection in first biopsy and repeat biopsy after negative transrectal ultrasound-guided biopsy: The Victorian Transperineal Biopsy Collaboration experience. BJU international 2015;116:568-76.

8. Scott S, Samaratunga H, Chabert C, Breckenridge M, Gianduzzo T. Is transperineal prostate biopsy more accurate than transrectal biopsy in determining final Gleason score and clinical risk category? A comparative analysis. BJU international 2015;116:26-30.

9. Udeh EI, Amu OC, Nnabugwu II, Ozoemena OFN. Transperineal versus transrectal prostate biopsy: Our findings in a tertiary health institution. Nigerian journal of clinical practice 2015;18:110-4.

10. Krughoff K, Eid K, Phillips J, et al. The accuracy of prostate cancer localization diagnosed on transrectal ultrasound-guided biopsy compared to 3-dimensional transperineal approach. Advances in urology 2013.

11. Kawakami S, Hyochi N, Yonese J, et al. Three-dimensional combination of transrectal and transperineal biopsies for efficient detection of stage T1c prostate cancer. International journal of clinical oncology 2006;11:127-32.

12. Shida Y, Hakariya T, Takehara K, Onita T, Miyata Y, Sakai H. Comparison between a combined transrectal and transperineal approach and a transrectal approach for prostate rebiopsy. Anticancer Research 2016;36:4685-90.

13. Paul Whelan J, Chin JL, Shapre JR, Davis IR. Transrectal needle aspiration versus transperineal needle biopsy in diagnosis of carcinoma of prostate. Urology 1986;27:410-4.

14. Grummet JP, Weerakoon M, Huang S, et al. Sepsis and 'superbugs': Should we favour the transperineal over the transrectal approach for prostate biopsy? BJU international 2014;114:384-8.

15. Miller J, Perumalla C, Heap G. Complications of transrectal versus transperineal prostate biopsy. ANZ journal of surgery 2005;75:48-50.

16. Dieffenbacher SC, Popeneciu IV, Radtke JP, et al. Diagnostic accuracy of transperineal MRI fusion biopsy in comparison to transrectal biopsy with regard to incidental histopathological findings in transurethral resection of the prostate. Urologia Internationalis 2017;99:162-7.

17. Tewes S, Peters I, Tiemeyer A, et al. Evaluation of MRI/Ultrasound Fusion-Guided Prostate Biopsy Using Transrectal and Transperineal Approaches. Biomed Res Int 2017;2017:2176471.

18. Namekawa T, Fukasawa S, Komaru A, et al. Prospective evaluation of the safety of transrectal ultrasound-guided transperineal prostate biopsy based on adverse events. International journal of clinical oncology 2015;20:1185-91.

19. Miano R, De Nunzio C, Kim FJ, et al. Transperineal versus transrectal prostate biopsy for predicting the final laterality of prostate cancer: Are they reliable enough to select patients for focal therapy? Results from a multicenter international study. International Braz J Urol 2014;40:16-22.

20. Hara R, Jo Y, Fuji T, et al. Optimal approach for prostate cancer detection as initial biopsy: Prospective randomized study comparing transperineal versus transrectal systematic 12-core biopsy. Urology 2008;71:191-5.

21. Takenaka A, Hara R, Ishimura T, et al. A prospective randomized comparison of diagnostic efficacy between transperineal and transrectal 12-core prostate biopsy. Prostate cancer and prostatic diseases 2008;11:134-8.

22. Cerruto MA, Vianello F, D'Elia C, Artibani W, Novella G. Transrectal versus transperineal 14-core prostate biopsy in detection of prostate cancer: a comparative evaluation at the same institution. Archivio italiano di urologia, andrologia : organo ufficiale [di] Societa italiana di ecografia urologica e nefrologica 2014;86:284-7.

23. Guo LH, Wu R, Xu HX, et al. Comparison between Ultrasound Guided Transperineal and Transrectal Prostate Biopsy: A Prospective, Randomized, and Controlled Trial. Scientific reports 2015;5.

24. Emiliozzi P, Corsetti A, Tassi B, Federico G, Martini M, Pansadoro V. Best approach for prostate cancer detection: A prospective study on transperineal versus transrectal six-core prostate biopsy. Urology 2003;61:961-6.

25. Watanabe M, Hayashi T, Tsushima T, Irie S, Kaneshige T, Kumon H. Extensive biopsy using a combined transperineal and transrectal approach to improve prostate cancer detection. Int J Urol 2005;12:959-63.

26. Abdollah F, Novara G, Briganti A, et al. Trans-rectal versus trans-perineal saturation rebiopsy of the prostate: Is there a difference in cancer detection rate? Urology 2011;77:921-5.

27. Tian X, Zhu C, Li T, Li X. Comparison of the clinical value of transperineal and transrectal prostate biopsy guided by transrectal ultrasonography in diagnosis of prostate cancer. China Journal of Modern Medicine 2014;24:80-2.

28. Yuan L-r, Zhang C-g, Lu L-x, et al. Comparison of ultrasound-guided transrectal and transperineal prostate biopsies in clinical application. Zhonghua nan ke xue = National journal of andrology 2014;20:1004-7.

29. Pepe P, Garufi A, Priolo G, Pennisi M. Transperineal Versus Transrectal MRI/TRUS Fusion Targeted Biopsy: Detection Rate of Clinically Significant Prostate Cancer. Clinical genitourinary cancer 2017;15:e33-e6.

30. Di Franco CA, Jallous H, Porru D, et al. A retrospective comparison between transrectal and transperineal prostate biopsy in the detection of prostate cancer. Archivio Italiano di Urologia e Andrologia 2017;89:55-9.
