# Supplementary figures and images for: Transperineal versus transrectal prostate biopsy in the diagnosis of prostate cancer: a systematic review and meta-analysis
Source: World J Surg Oncol. 2019 Feb 13;17:31. doi: 10.1186/s12957-019-1573-0 (PMC6375152; doi:10.1186/s12957-019-1573-0)

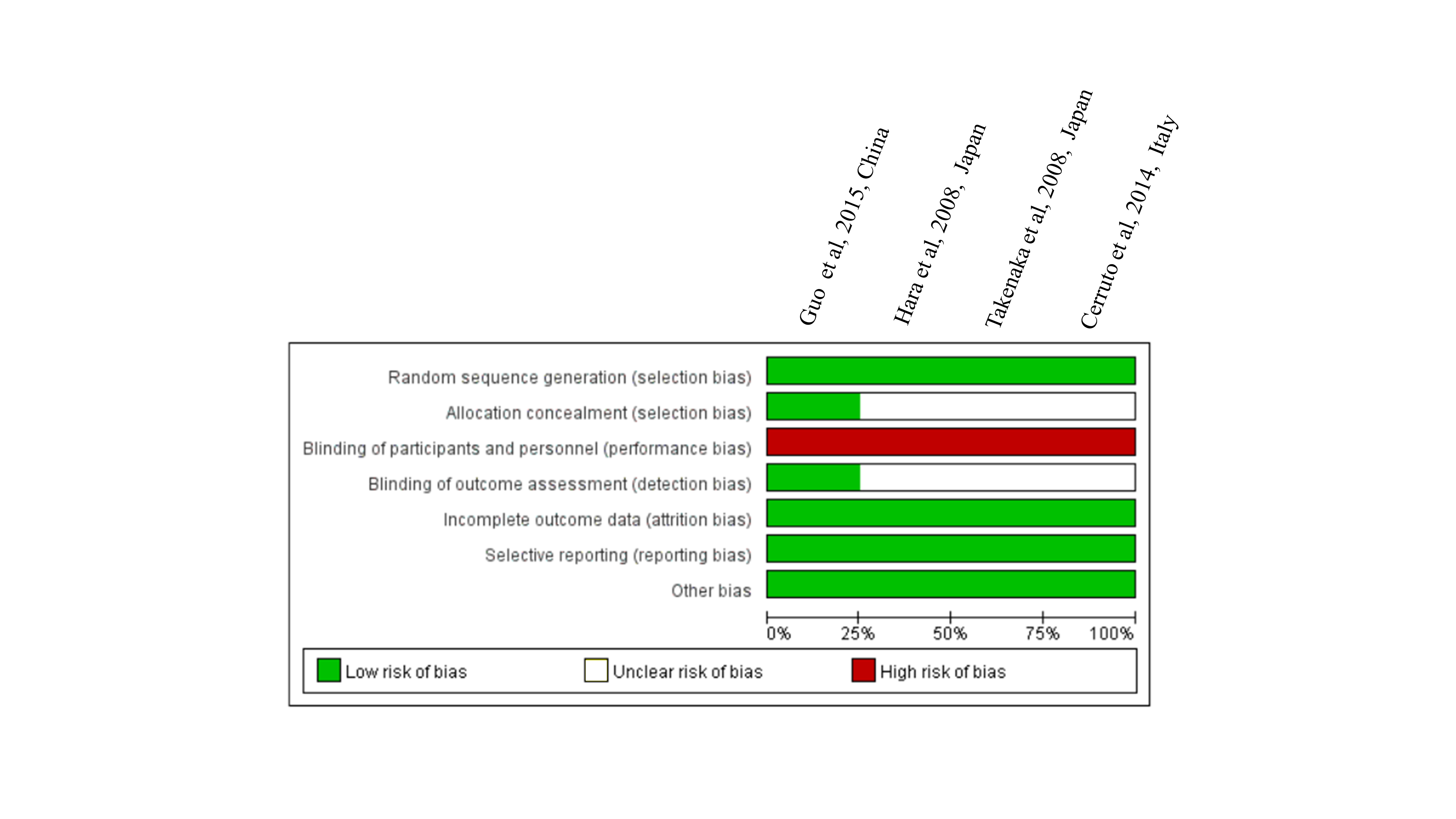

Supplement: Supplementary file 1 — Table S1. Details of excluded studies. Table S2. Sensitivity analysis of RCTs. Table S3. Sensitivity analysis of observational studies. Figure S1. Risk of bias assessment of RCTs. (ZIP 605 kb) [file 12957_2019_1573_MOESM1_ESM.zip › Figure S1.tif]
